# Friends in All the Green Spaces: Weather Dependent Changes in Urban Mosquito (Diptera: Culicidae) Abundance and Diversity

**DOI:** 10.3390/insects12040352

**Published:** 2021-04-15

**Authors:** Heli Kirik, Viktoria Burtin, Lea Tummeleht, Olavi Kurina

**Affiliations:** 1 Inst of Agricultural and Environmental Sciences, Estonian University of Life Sciences, Friedrich Reinhold Kreutzwaldi 5D, 51006 Tartu, Estonia; olavi.kurina@emu.ee; 2 Environmental Board, Narva mnt. 7a, 15172 Tallinn, Estonia; viktoria.burtin@keskkonnaamet.ee; 3 Inst of Veterinary Medicine and Animal Sciences, Estonian University of Life Sciences, Friedrich Reinhold Kreutzwaldi 62, 51006 Tartu, Estonia; lea.tummeleht@emu.ee

**Keywords:** *Aedes*, *Anopheles*, *Coquillettidia*, *Culex*, *Culiseta*, entomology, Estonia, environment, pathogen vectors

## Abstract

**Simple Summary:**

Many female mosquitoes require vertebrate blood for egg production. Cities are becoming increasingly important points of contact between mosquitoes and their prey, as large-scale urbanization continues. Human settlements represent unique but fragmented habitats that are permanently warmer than rural areas. Because of this, there is a growing demand to better understand urban mosquito populations and the factors affecting them in various circumstances. The aim of this study was to investigate the weather conditions influencing mosquito species and abundance in a Northern European town. Thus, a three-year-long mosquito collection effort was undertaken in Estonia. Results indicated that the number of active mosquitoes decreased with wind and higher temperatures. Interestingly, there was a significant negative correlation between temperature and humidity. Furthermore, while mosquitoes belonging to the *Culex pipiens*/*Culex torrentium* group were consistently abundant during the end of the warm season, other dominant species varied considerably between the months and the three study years. Overall, springtime hydrological conditions seemed to greatly influence the mosquito season. Urbanization could generate both higher temperatures and drier environments, resulting in fewer mosquitoes in some areas. This study also revealed the mosquito species most likely to contribute to disease transmission in Estonian towns.

**Abstract:**

Mosquitoes (Diptera: Culicidae) are universally recognized as troublesome pests and vectors of various pathogens and parasites. Understandably, the species makeup and diversity of individual populations depends on local and broad scale environmental trends, especially on temperature and hydrological variations. Anthropogenic landscapes make for unique habitats, but their effect on insects likely varies across climatic regions. The aim of this study was to investigate the diversity and seasonal patterns of urban mosquitoes in the boreal region. Specimens were collected with an insect net from May to September during three years and determined to species or species group level. Weather information was added to each data point and results analyzed using multivariate regression models. Fieldwork yielded 1890 mosquitoes from four genera. Both abundance and the effective number of species (ENS) significantly decreased during the study period. The number of collected mosquitoes had a negative correlation with wind speed and temperature, latter of which exhibited a negative association with humidity. Species succession followed predictable patterns, but with some variation between years. Still, *Culex pipiens*/*Culex torrentium* were the most abundant throughout the study. Importantly, all dominant species were known disease vectors. Our work showed that higher temperatures could result in fewer mosquitoes in boreal towns.

## 1. Introduction

Mosquitoes (Diptera: Culicidae) are common biting insects found on almost every continent: Thus far, a total of 3583 species have been recorded from various parts of the world [1,2]. Moreover, mosquitoes are the primary transmitters, also known as vectors, for many of the most important arthropod-borne diseases [3]. All vector-borne diseases combined account for about 17% of the global disease burden, currently endangering around 80% of the world’s population [4]. However, ongoing processes like urbanization, alterations in agricultural practices, deforestation, climate change as well as socioeconomic developments influence the prevalence and geographic ranges of both vectors and vector-borne pathogens [5,6,7]. Furthermore, different mosquito species can act as the principal vector for the same pathogen depending on whether the transmission cycle takes place in a sylvatic, rural or urban setting [8,9]. Hence, it is not only important to study global mosquito diversity patterns, but to also understand how mosquito communities and vector-human interactions are shaped by local conditions, as mosquitoes are the most likely vectors to cause vector-borne disease epidemics in urban environments [10].

Human habitats present mosquitoes with unique challenges: densely populated areas provide human-biting insects with a reliable food source, giving anthropophilic species a notable evolutionary advantage [11]. Furthermore, cities present a highly fragmented setting, where biodiversity is influenced by the interactions between the microenvironment and urbanization specific broad-scale trends [12,13,14]. For example, the urban heat island effect is a well-established phenomenon: Large settlements tend to be significantly warmer than the surrounding areas [15,16,17]. These higher temperatures help create suitable habitats for organisms normally found in lower latitudes, supporting the spread and establishment of invasive species [18,19]. This in turn allows for the northward expansion of exotic vector-borne pathogens, exposing more people to the risk of infections [5,20]. On the other hand, urbanization is most commonly associated with a general decrease in species diversity, affecting specialized organisms more than generalists, although this varies by taxon [12,13,18]. Densely populated settlements naturally contribute to the abundance and development of synanthropic organisms. Therefore, it is to be expected that anthropogenic landscapes favor some mosquito species above others [11,21]. Urban green spaces are particularly noteworthy for providing mosquitoes with ample shelter and a variety of food sources [22,23]. Hence, as mosquitoes can be a severe nuisance as well as present a clear health risk, it is important to develop a better understanding of their community structure in various locations with differing levels of urban development [5,21,24].

Biodiversity, species abundance and the community makeup of anthropogenic landscapes has received increasing attention in the past decades [25]. A number of studies have investigated general mosquito abundance and diversity in various towns and suburban areas as well as how these populations respond to different weather conditions [26,27,28,29]. Others have examined the urban lifecycles of the most common or significant synanthropic mosquito species [30,31]. Some studies have concentrated on the ways the characteristics of urban green spaces can influence mosquito abundance, regardless of weather patterns, and how these areas could be designed to be safer for humans [32,33,34]. Similarly, efforts have been made to improve methods of detecting areas which serve as mosquito refuges and breeding sites [35]. For example, previous research has shown that the container breeding *Culex* (*Cx.*) *pipiens* Linnaeus, 1758 is exceedingly common and abundant in urban environments [26,27]. Nonetheless, in the Po Plain Valley region of Italy it was found that during summer months the overall density of *Cx. pipiens* was still higher in rural sites rather than urban areas [30]. Furthermore, field tests in Thailand indicated that environmental characteristics like closeness of waterbodies and forested areas as well as higher canopy cover increased the number of larvae predators in mosquito breeding sites, but these predators attacked mosquitoes of various species at different rates [34]. Researchers looking at city parks in Manaus, Brazil collected mosquitoes from various distances from the forest edge, revealing a significant difference in the species composition of sites near the perimeter and those 500 m into the forest [36]. On the other hand, a study conducted in Hong Kong found that while temperature had an overarching effect on urban mosquito populations, windiness had a negative effect on mosquito abundance in rooftop green spaces, making these areas safer for residents than ground level parks [33]. Research in Chicago, IL, USA demonstrated that species richness as well as diversity correlated positively with habitat heterogeneity, and climatic variability appeared to influence mosquito diversity patterns across the study sites [14]. In the same study, doctor Chaves and colleagues also found that an increase in species diversity coincided with a reduction in mosquito abundance. All in all, it is clear that urban mosquito populations are shaped by both largescale progresses as well as the local microhabitat, but the nature and strength of these interactions should be further examined in settlements with various levels of urbanization and in different climate zones [12].

Thus far, most studies regarding urban mosquitoes have been conducted in the tropics, subtropics and the warmer areas of the temperate climate zone. The aim of this study was to better understand the main factors influencing mosquito abundance and species diversity in the urban green spaces of a low density settlement in the boreal biome. For this purpose, four main hypotheses were established:

1. Higher temperature and relative humidity values result in a greater number of active mosquitoes.
2. Stronger winds are expected to have a negative correlation with the number of active individuals.
3. The ratio of collected female and male mosquitoes varies over the warm season, because male mosquitoes have shorter lifespans [37] and thus their abundance should be more sensitive to recent adverse weather conditions.
4. Urban mosquito populations are dominated by one or two abundant synanthropic species.

## 2. Materials and Methods

This study was conducted in Tartu, the second largest town in Estonia, situated on the east and west shores of river Emajõgi. Weather in Estonia is characterized by the temperate continental climate with cold winters and brief but warm summers according to the Köppen-Trewartha climate classification system [38,39]. The European Commission considers Estonia to belong to the Boreal biome [40]. Tartu itself is a university town with slightly more than 96,000 inhabitants (624.2 inhabitants per km^2^) and serves as the regional center for Southern-Estonia [41]. The town area spans 38.80 km^2^: This includes 3.90 km^2^ (about 10.1%) of urban green spaces and 5.10 km^2^ (13.1%) of natural vegetation [42,43].

**Figure 1:**
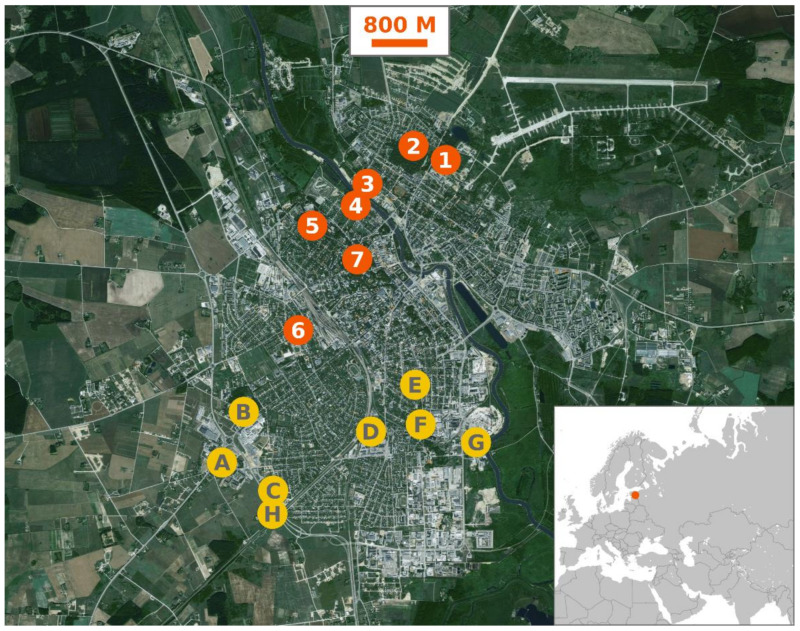
Map showing the collection sites in Tartu and the location of Estonia. Sites 1, 2, 3, 4, 5 and 6 were used in 2013, 2016 and 2017. Site 7 was included in the study in 2016 and 2017. Sites A, B, C, D, E, F, G and H were added in 2017. Base map of Tartu: Estonian Land Board (https://xgis.maaamet.ee/xgis2/page/app/maainfo, accessed on 18 November 2020), 2019. Map of Europe: © MapTiler; © OpenStreetMap contributors (https://www.maptiler.com/, accessed on 18 November 2020).

Mosquitoes were collected using a 50 cm diameter mesh net once a week from May to October during 2013, 2016 and 2017. Hand-net collections have been previously used by numerous researchers [44] and this method was chosen for its cost effectiveness as well as robustness, as it allowed collecting mosquitoes from busy areas where the use of stationary traps was not possible. Collection sites (Figure 1) were visited each week in a changing order starting from five o’clock in the afternoon. All in all, six collection sites were sampled during 2013, one new site was added in 2016 and further eight sites were added in 2017. Collection sites were located in the shaded areas of parks, near play areas, recreational trails or footpaths:

- Site 1 (58°23′40.6″ N, 26°44′05.6″ E) was situated in a corner of an abandoned gravel quarry by a well-traveled park with large trees but very little brush.
- Site 2 (58°23′44.6″ N, 26°43′44.4″ E) was in a sitting area in the town’s largest commentary complex, surrounded by both old trees as well as ornamental hedges.
- Site 3 (58°23′24.7″ N, 26°42′55.7″ E) was located on the north shore of river Emajõgi, under sparse old trees.
- Site 4 (58°23′20.1″ N, 26°42′52.6″ E) was situated on the south side of river Emajõgi and included both old park trees as well as brush.
- Site 5 (58°23′05.5″ N, 26°42′19.7″ E) was in Tähtvere park by a large ornamental bush, sparsely surrounded by old trees.
- Site 6 (58°22′17.8″ N, 26°41′58.1″ E) was in Mathieseni park, on the south side of a row of tall ornamental bushes, surrounded by park trees. This park borders the Tartu University Hospital and is visited by both faculty and patients.
- Site 7 (58°22′52.10″ N, 26°42′49.13″ E) was situated on an uneven natural hill called Toomemägi, close to the ruins of a former cathedral. This park is dotted by trees and the irregular features as well as ruined structures offer plenty of shade.
- Site A (58°21′13.4″ N, 26°40′45.5″ E) was located in a tree enclosed green space at the edge of the town.
- Site B (58°21′36.9″ N, 26°41′10.4″ E) was on the border between single-family homes and a small densely wooded area.
- Site C (58°21′1.3″ N, 26°41′30.6″ E) was situated beside a construction site at the edge of the town, with very few trees or bushes in the vicinity.
- Site D (58°21′26.4″ N, 26°42′60.0″ E) was on the margins of Pauluse cemetery, which is dotted by old trees and features a small pond.
- Site E (58°21′50.6″ N, 26°43′43.0″ E) was situated close to the border between the yard of St. Alexander’s Orthodox Church and surrounding residential buildings.
- Site F (58°21′36.5″ N, 26°43′56.3″ E) was located in a small parking area surrounded by Forseliuse park, which feature large trees and a children’s play area.
- Site G (58°21′23.8″ N, 26°44′31.7″ E) was on a construction site near river Emajõgi, surrounded by large commercial buildings.
- Site H (58°20′52.9″ N, 26°41′37.3″ E) was located in a sparsely populated area near the city limits, overgrown with brush.

The collection protocol called for two times 25 swings with the insect net and specimens were gathered between as well as after the sets with an aspirator. Net swings were made in the air and through the tips of soft vegetation. Date, time and the person collecting mosquitoes was recorded at each site. Mosquitoes were later killed by freezing and stored in 75% ethanol (C_2_H_5_OH) or as dry material at −20 °C. Specimens were identified to species or a species group level under a stereomicroscope Olympus SZ61 (Olympus Corporation, Shinjuku, Tokyo, Japan) using a standard taxonomic key [37] and their gender was recorded. Mosquitoes too damaged for identification were marked as “unspecified”. Afterwards, weather information was added to the data from the records of the Estonian Weather Service, based on the date and time of fieldwork. Data was acquired from Tartu-Tõravere meteorological station (58°15′51″ N, 26°27′41″ E), which is situated about 16 km southwest (SW) of the city limits of Tartu. Each catch in the dataset was provided with the measurements of time to sundown (min), temperature (°C), relative humidity (%), wind speed (m/s) and atmospheric pressure at sea level (hPa).

Shannon diversity indices (*H*) were calculated based on the number of mosquitoes and the quantity of different species in each catch [45]. The Shannon diversity index can be written as the following equation:

$$H=-\overset{S}{\underset{i=1}{\sum }}p_{i}\ln p_{i}$$

where *S* is the number of different mosquito species and *p* is the number of individuals of the same species divided by the number of all individuals. From this, the true diversity of the collected mosquito samples was calculated using the effective number of species (*ENS*). This statistic indicates what kind of a population with equally represented species the examined sample is similar to [46]. *ENS* was calculated by taking the exponential of the Shannon diversity index and the results were rounded to integers:

$$ENS=\exp\left(-\overset{S}{\underset{i=1}{\sum }}p_{i\;}\ln p_{i}\right).$$

As the mosquito count data had a Poisson distribution, the parameter lambda (λ) was used to represent the average number of mosquitoes caught during collection events. For the same reasons, 95% confidence interval (CI), instead of standard deviation, was used to characterize dispersion. Additional statistical analyses were done in the free software R version 3.6.1 [47]. Mosquitoes which could not be identified to species or species group level were only included in the dataset when analyzing specimen yields and removed when examining species diversity. Additionally, data was cleaned of outliers and the independent variables were checked for pairwise correlations using R package “psych” [48]. The degree of correlation was evaluated using the non-parametric Kendall rank correlation coefficient (τ). As relative humidity and time until sunset were moderately correlated with each other (τ = −0.43) as well as the month (τ = 0.45 and τ = −0.44, respectively) they were dropped from the analysis. As no fieldwork was done in October in 2013 and only few catches were made during that month in 2016, the records for October were also eliminated from the dataset. Days when fieldwork was terminated early due to rainfall were removed.

Using the R package “MASS” [49], negative binomial generalized linear model (GLM) was employed to determine the character and power of the relationship between the independent variables (collection site, month and year of collection, temperature, wind speed, relative humidity and gender) and the number of collected mosquitoes. This was due to the dataset exhibiting both over dispersion and zero inflation. On the other hand, a GLM with Poisson distribution was used for modeling the relationships between independent variables and ENS. Non-significant variables were removed from the models by hand. Models were tested for over- and under-dispersion as well as zero inflation using the R packages “DHARMa” [50] and “performance” [51], respectively. Furthermore, the R package “mctest” [52,53] was employed to evaluate the level of multicollinearity among the independent variables based on variance inflation factor (VIF) and tolerance (TOL). Illustrative figures were generated using the R package “ggplot2” [54]. When necessary, correlation statistics included on these figures were calculated by conducting a non-parametric test using Kendall rank correlation.

## 3. Results

The dataset analyzed in this study consisted of 1890 mosquitoes caught from 15 collection sites in the town of Tartu: 654 mosquitoes were collected in 2013 (74.01% of these were female), 556 in 2016 (53.60% female) and 680 in 2017 (58.97% female). Of these individuals, 47 mosquitoes were too damaged to be identified by their morphological traits. It should be stressed, that in 2013 six collection sites were sampled, in 2016 one new site was added and in 2017 a total of eight additional sites were added. Therefore, while the total number of collected mosquitoes was similar between the three years, in reality the mean number of individuals caught during each collection event decreased from 6.41, 95% CI [6.22–6.61] (Poisson lambda (λ), 95% confidence interval (CI) [lower limit–upper limit]) in 2013 to 3.78, 95% CI [3.62–3.94] in 2016 and 2.53, 95% CI [2.41–2.65] in 2017. The number of mosquitoes caught during one collection event varied from zero to 90 and was influenced by year, month, temperature, wind conditions, insect gender as well as study site, but also by the associations between these factors (Table 1). Species diversity, represented by the effective number of species (ENS), also showed a slight decrease between the three years: the average ENS was 1.59, 95% CI [1.40–1.78] in 2013, 1.39 [1.23–1.55] in 2016 and 1.11 [0.99–1.23] in 2017. Additionally, the ENS of a single collection event only varied from zero to six and was influenced by the collection year and site (Table 2).

**Figure 2:**
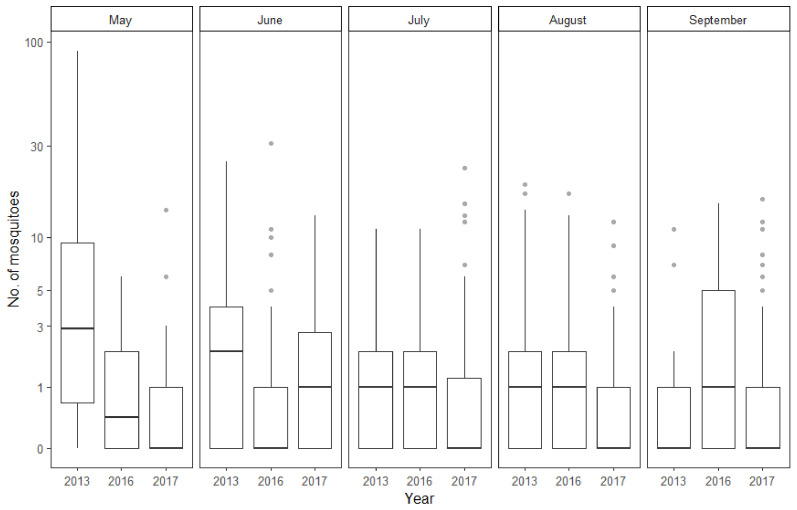
Average number of mosquitoes collected during the different months of the study period. Boxplots showing the median (dark line dividing the box), interquartile range (IQR) containing 50% of the data points (length of the box), upper and lower quartiles (whiskers) and outliers (gray dots). *Y*-axis has been transformed to a logarithmic scale for ease of viewing.

The number of collected mosquitoes was dependent on the collection year and month as well as on the interaction between the two variables (Figure 2). On average, mosquito collection events yielded far more individuals during 2013 than during 2016 and 2017. However, there was also marked variance between the fieldwork months. All in all, higher numbers of mosquitoes were caught during May and June. Noticeably fewer mosquitoes were collected on average during July, August and September. Interestingly, when looking at how the interactions between year and month influence average mosquito yield, it seems that in 2016 and 2017 the average number of mosquitoes collected during May is significantly smaller than in 2013 compared to the other months. Because of this, the interactions between the later years and other collection months, except for June in 2016, show a positive effect on the average mosquito yield.

**Figure 3:**
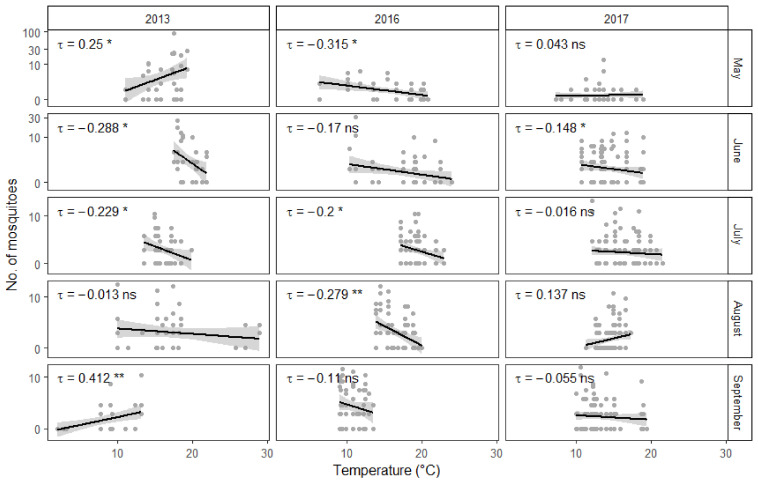
Influence of temperature on the abundance of mosquitoes during different months. Y-axis has been transformed to a logarithmic scale for ease of viewing. Gray points represent fieldwork results. Linear regression lines are surrounded by gray areas representing 95% confidence intervals. Correlation statistics have been calculated using the non-parametric Kendall rank correlation. Significance: >0.05 = “ns”, 0.05 to 0.01 = “*”, 0.01 to 0.001 = “**”.

**Figure 4:**
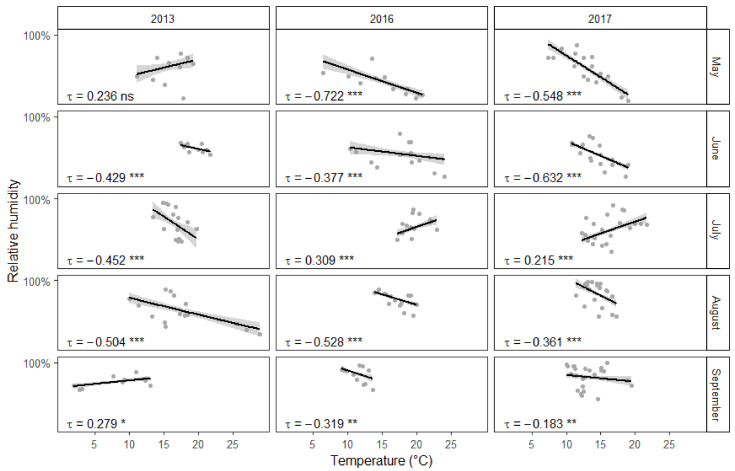
Correlation between temperature and relative humidity. On many occasions higher temperatures correlated with lower relative humidity. Collection events are represented by gray dots, linear regression lines are surrounded by gray areas representing 95% confidence intervals. Correlation statistics have been calculated using the non-parametric Kendall rank correlation. Significance: >0.05 = “ns” 0.05 to 0.01 = “*”, 0.01 to 0.001 = “**”, <0.001 = “***”.

Somewhat surprisingly, higher temperatures appeared to correlate with fewer collected mosquitoes (Figure 3). Interestingly, there was a negative association between temperature and relative humidity (Figure 4). On the other hand, as could be expected, stronger winds in the area of the town resulted in fewer mosquitoes being collected during fieldwork. However, there was no significant interaction between individual study sites and general wind conditions. Quite predictably, male mosquitoes were collected much less often than females. Additionally, there appears to be an interaction between collection month and insect gender. The proportion of males among the collected mosquitoes was overall significantly larger in August than in May. This difference becomes even more pronounced in September. However, there was no significant interaction between collection year and gender. Furthermore, some of the 15 study sites yielded more mosquitoes on average than others.

**Figure 5:**
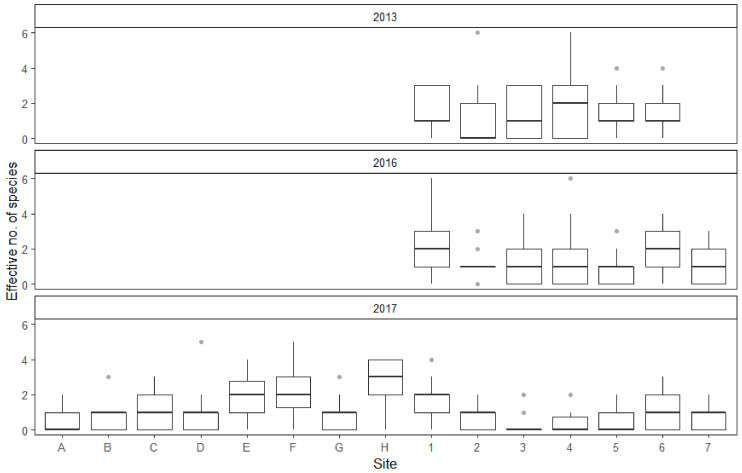
Average effective no. of species (ENS) of each collection site throughout the three study years. Boxplots showing the median (dark line dividing the box), interquartile range (IQR) containing 50% of the data points (length of the box), upper and lower quartiles (whiskers) and outliers (gray dots).

The effective number of species (ENS) statistic was chosen to represent population diversity. ENS was calculated for every collection event, based on all of the mosquitoes that could be identified to species or species group level by morphological markers. Results show that the diversity of the collected individuals was influenced by both collection site and year (Figure 5). ENS did not appear to be influenced by the study month, temperature, wind conditions or atmospheric pressure. Furthermore, some collection sites yield more mosquito species on average than the reference site. As with mosquito abundance, the average effective number of species decreased from 2013 to 2017. Interestingly, the overall number of recorded species actually increased during the study.

**Figure 6:**
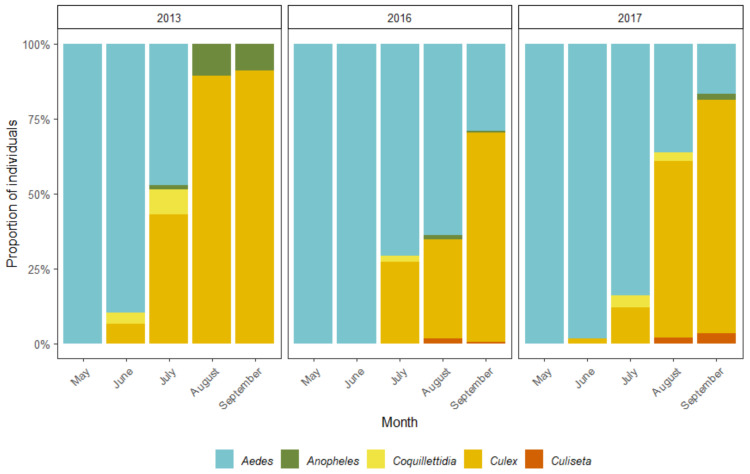
The succession of mosquitoes from different genera over the study period. The percentage of mosquitoes from five different genera collected in 2013, 2016 and 2017, showing the transition from *Aedes* to *Culex* dominated populations during the warm months.

All in all, 20 different mosquito species and species groups from five genera (*Aedes* Meigen, 1818, *Anopheles* Meigen, 1818, *Coquillettidia* Dyar, 1904, *Culex* Linnaeus, 1758 and *Culiseta* Felt, 1904) were collected during the study period—14 species in 2013 and 17 in both 2016 as well as 2017. There are thought to be about 32 mosquito species in Estonia [55]. *Culex* (*Culex*) *pipiens* Linnaeus, 1758 together with *Cx.* (*Culex*) *torrentium* Martini, 1925 were the most collected mosquitoes during all three study years. However, the abundance of other common species varied dramatically from year to year (Table 3). Furthermore, the dominant species changed within each study year as the warm season progressed (Figure 6). In 2013, the species or species group most commonly collected in May was *Ae*. *communis* (de Geer, 1776), and in June *Ae*. *annulipes* group. *Cx*. *pipiens/ Cx*. *torrentium* were dominant throughout the remaining warm season. On the other hand, the years 2016 and 2017 were similar to each other. In both years *Ae*. (*Ochlerotatus*) *punctor* (Kirby, 1837) together with *Ae*. (*Ochlerotatus*) *punctodes* (Dyar, 1922) and *Ae*. *annulipes* group dominated in May and June, respectively. *Cx*. *pipiens/Cx*. *torrentium* group as well as the *Ae*. *cinereus*/*Ae*. *geminus* group were most numerous in July and August. As expected, *Cx*. *pipiens/Cx*. *torrentium* were the predominant individuals in September.

## 4. Discussion

The chosen collection method only allowed to capture a relatively small sample, 1890 individuals, of the local mosquito population, but this was sufficient to illustrate how different environmental factors influence the quantity and diversity of active mosquitoes in urban green spaces. Results show that both the mean number of collected mosquitoes as well as the average effective number of species decreased significantly from 2013 to 2017, while the number of recorded species actually increased. There have been no coordinated mosquito control efforts in Tartu, therefore the differences in abundance and variety are probably due to changes in annual weather patterns. Furthermore, it is likely that the town environment further amplified the effects of some of these atmospheric conditions.

Temperature, precipitation and humidity are considered to be the most important weather factors influencing mosquito abundance [5]. Additionally, these aspects are not only important during the warm season: weather conditions during winter and early spring can also have a profound effect on bloodsucking insects. For example, many of the spring mosquito species rely on snowmelt pools or flood waters for the development of the new generation and therefore require snowy winters [37]. In fact, this is the most likely explanation for why fieldwork in May 2013 yielded so many more mosquitoes on average compared to 2016 and 2017. According to the Estonian Weather Service, snow could be found everywhere in Estonia during the first three months of 2013, and the snow cover finally completely disappeared by the end of April, resulting in routine flooding [56]. On the other hand, snow conditions were less stable during the first months of 2016, with the snow partially melting many times during January, February and March, then finally disappearing at the beginning of April [57]. January and February of 2017 were especially warm and snow could only form thin layers on the ground, similar pattern continued in March with the snow cover melting and reforming many times until the end of the month [58]. Due to the absence of snow at the end of April during the last two study years, there was likely less floodwater available for the mosquito larvae that depend on it. Moreover, the mean temperature and the amount of rainfall in May also differed substantially from 2013 to 2017. May in 2013 was warm (mean temperature 2.9 °C higher than normal) as well as rainy (mean precipitation 22 mm higher than normal), while 2016 was warm (mean temperature 2.7 °C higher than normal) but dry (mean precipitation 24 mm less than normal) and 2017 was cool (mean temperature 1.1 °C colder compared to normal) as well as dry (mean precipitation 27 mm less than normal) [56,57,58]. Both winter snow cover as well as weather conditions in May likely played a significant role in the decrease of spring and early summer mosquitoes from 2013 to 2017. Furthermore, it has been previously reported, that the mean relative humidity in May can strongly influence the insect abundance throughout the rest of the warm season [30]. It is clear that mosquitoes started off with high abundance in 2013, but the number of individuals noticeably decreased over the rest of the warm season. However, the number of mosquitoes caught during collection events followed the complete opposite trajectory in 2016, when September yielded the most specimens. Furthermore, the 2017 study year proved the most variable as the number of collected mosquitoes was similarly low in May but also noticeably dropped in August. All this indicates that the variations in local weather conditions between years and months play an important role in the number of actively flying mosquitoes.

Undoubtedly, air temperature is an important factor in determining the development speed of mosquito larvae and air temperature often correlated with the number of mosquitoes collected during the three years of this study. However, warmer temperatures were somewhat surprisingly often associated with fewer captured mosquitoes. This could be explained by the negative correlation between temperature and relative humidity. Mosquitoes are relatively delicate insects and risk drying out in direct sunlight and low humidity conditions. Therefore it is not surprising that high relative humidity is positively correlated with higher numbers of active mosquitoes [59]. At the same time, urban environments have been shown to have lower relative humidity and higher temperature values than the surrounding areas [17,60]. This most likely means that the collection sites were in reality even warmer and drier than what the closest weather station measured. However, there were also exceptions to the general trend, when the relationship between temperature and the number of collected mosquitoes could not be explained by the level of relative humidity. Regrettably, this study fails to offer an alternative explanation to these cases. Finally, there was also a statistically important positive correlation between temperature and the number of active mosquitoes during September 2013, likely because temperatures had dropped below +5 °C, which had a noticeable negative impact on collection success. All of this taken together means that the first hypotheses postulated in this study is not completely correct. While both higher temperature and humidity values are favorable for mosquitoes, there can be a negative correlation between the two factors.

Wind conditions were also shown to affect mosquitoes and the second hypotheses of this study was proven correct. Stronger winds had a predictably negative correlation with the average number of individuals collected during fieldwork. Although moderate wind speeds can be helpful to mosquitoes by facilitating long distance dispersal and by carrying host scent further down-wind, many mosquito species have trouble flying in windy conditions [37,61,62]. For example, strong winds have been proposed to be the main reason why mosquitoes avoid inhabiting urban green roofs [33]. It should be noted that there was no significant interaction between wind speed and collection site in the current study, indicating that none of the sites were more protected from the wind than others. All in all, it could be advantageous to take wind conditions into consideration when deigning urban green spaces, in order to avoid creating areas which could become too shielded from the wind and facilitate mosquito biting activity.

Collecting mosquitoes with an insect net made it possible to capture both female and male individuals. Although only female mosquitoes require blood and thus act as disease vectors and pests, a better understanding of the male population is also necessary for the development of effective mosquito control measures [63]. Predictably, female mosquitoes were collected more often than males: Although many species exhibit a 1:1 sex ratio and some are even male biased [64], female mosquitoes were likely attracted to the person conducting fieldwork, while males were caught more randomly. However, there was also an interaction between mosquito gender and month. More male mosquitoes were collected in the last two months of the study period compared to May and this change was not paralleled by females. This could be explained in part because *Cx*. *pipiens* females, one of the most numerous species in August and September, do not usually take a blood meal before overwintering [37] and thus were less likely to be drawn to the fieldworker. On the other hand, it seems that yearly weather changes influence both genders similarly as there was no statistically significant interaction between mosquito gender and collection year. All in all, hypotheses number three of this study was not proven correct. Although the sex ratio of the collected mosquitoes varied over the fieldwork period, these differences were better explained by other factors than weather fluctuations during the warm season.

Some collection sites yielded significantly more mosquitoes on average than the reference site A. Out of the six spots that were visited during all of the collection years, more mosquitoes were caught at sites 1 and 6. Yet, this result cannot be explained by the factors accounted for in this study. There was no discernable interaction between collection sites and wind. Neither do sites 1 and 6 noticeably differ from the others in the availability of mosquito breeding sites. Furthermore, sites C, D, E, F, G and H also yielded significantly more mosquitoes on average compared to the reference site. However, care should be taken when comparing sites A through H to sites 1 to 7, as these were collected from by different people and collector bias cannot be excluded. Finally, there are also landscape factors that can influence mosquito abundance and help explain the variation between collection sites [32], but these are outside the scope of this study.

The average effective number of species (ENS) was only significantly different between 2013 and 2017, with the fieldwork results of the later year displaying less species diversity. This was most likely due to the 2017 study year yielding fewer collected mosquitoes in general and normally rare species were even less likely to be caught. This was further reflected in fact that the collection sites E, F, H, 1 and 6, which had some of the best mosquito yields on average, were also the ones with the highest mean ENS results. Although, there were some differences: sites G and D did not appear to have a statistically significant effect on ENS, but exhibited a larger positive effect on the average number of collected mosquitoes than sites 1 and 6. Hence, there likely is some variation in species diversity between the collection sites that cannot be explained by a larger number of sampled individuals.

Species succession throughout the warm season follows predictable patterns. *Ae*. *communis*, *Ae*. *intrudens* and *Ae*. *punctor*/*Ae*. *punctodes* are snowmelt mosquitoes able to tolerate colder conditions [37] and thus were understandably most numerous in May. Interestingly, while *Ae*. *communis* and *Ae*. *intrudens* was exceedingly numerous in 2013, they were much less prominent in the later study years. *Ae*. *intrudens* in particular was almost absent in 2017. While the number of collected *Ae*. *punctor*/*Ae*. *punctodes* individuals also fell during 2016 and 2017, the change was much less dramatic. Mosquitoes from the *Ae*. *annulipes* group and *Ae*. *cinereus*/*Ae*. *geminus* also appeared during the beginning of the warm season, overtaking snowmelt mosquitoes as the most numerous species in June and July, with some variation between the years. Individuals of the *Ae*. *annulipes* group could be found from May to August but were most numerous in June. However, in 2013 the largest number of *Ae*. *cinereus*/*Ae*. *geminus* mosquitoes were collected in May, but no individuals from these species could be found in August or September. On the other hand, in the later study years *Ae*. *cinereus*/*Ae*. *geminus* were much more numerous during the summer months and could still be collected throughout September. *Ae*. *cinereus* and *Ae*. *geminus* are thought to prefer semi-permanent water features, but also require warmer temperatures than the snowmelt mosquitoes [37]. Therefore, May being both warm and rainy in 2013 most likely explains why these species were active early in the season at the time, but suitable breeding sites likely dried up over the summer. *Cx*. *pipiens* and *Cx*. *torrentium* were the most enduringly abundant species during the study period: every year first individuals started appearing at the beginning of summer and became dominant in September. Other various species were caught in low numbers, mostly over the summer months. All in all, it appears that the fourth hypothesis of this study was proven correct: the synanthropic species *Cx*. *pipiens* was abundant during every year of the study, while a few other species were dominant in some years but not others. Additionally, the 20 species collected in this study constitute about 62.5% of the overall mosquito richness in Estonia. Compared to the Estonian checklist, which was compiled in 1955 [65] and updated in 2014 [55], the urban mosquito fauna of Tartu is missing halophilic species as well as some species from the genera Culiseta. Some of the rarer *Aedes* species of Estonia were also not collected during this study. Still, the relatively high level of collected species indicates that the urban green spaces of Tartu encompass various microhabitats able to support both synanthropic and sylvan mosquitoes. Such environmental variety is commendable from a general biodiversity perspective but may also imply that it is more likely for future invasive mosquito species to become locally established.

Furthermore, the species most numerous in Tartu are all known disease vectors. For example, prior studies have identified *Cx*. *pipiens* and/or *Cx*. *torrentium* individuals infected with the West Nile virus [66,67], Ockelbo virus [68], Usutu virus [69], *Borrelia* (*B.*) *garinii* [70], *Francisella* (*F.*) *tularensis* [71], *Dirofilaria* (*D*.) *repens* and *D*. *immitis* [72,73]. Moreover, *Cx*. *pipiens* s.l. can play a key role in transferring pathogens between birds and humans [74]. *Ae*. *cinereus* mosquitoes have been associated with the Jamestown Canyon virus [75], Ockelbo virus [68], both *B. afzelii* and *B*. *garinii* [70], *F. tularensis* [71] as well as *D. repens* and *D*. *immitis* [76,77]. Lastly, the different species of the *Ae*. *annulipes* group have also been previously indicated in the transmission of *F. tularensis* [71] and *D*. *repens* [77].

Future work could sample both urban and rural habitats for comparison. Furthermore, establishing collection points in private yards would permit the use of stationary passive insect traps without losing the ability to collect male mosquitoes. These measures would give a more granular overview of how various conditions influence the changes in mosquito abundance and diversity.

## 5. Conclusions

The numbers of active mosquitoes inhabiting urban green spaces in the town of Tartu are greatly influenced by the variations in yearly weather patterns. The mean number of collected mosquitoes sharply declined between 2013 and 2017. This could have been in large part because of the winter snow conditions and the meteorological character of the end of spring, as May 2013 was set apart of the other study years by an abundance of snowmelt water as well as a warm and rainy weather. The number of active mosquitoes was also influenced by temperature, humidity and wind. Importantly, there was an apparent negative correlation between temperature and humidity, something that the urban environment most likely further enforced. This means that higher temperatures in the urban environments of the boreal biome may in some cases actually result in fewer active mosquitoes. Furthermore, stronger winds also decreased the number of collected mosquitoes. This is something that could be taken into account when planning for new urban parks with less mosquito biting activity. On the other hand, the diversity index of the collected mosquitoes, represented by the effective number of species (ENS), also declined from 2013 to 2017. This was most likely in part a side effect of the general degrease in the mean number of collected mosquitoes. All in all, *Cx*. *pipiens* together with *Cx*. *torrentium* remained the most abundant mosquito species throughout the three study years. Other dominant species tended to vary between the years. Worryingly, the most numerous species collected in this study are all capable of carrying several pathogens. In the light of ongoing anthropogenically driven environmental changes, the surveillance of mosquitoes as well as vector-borne pathogens is becoming increasingly necessary in colder climate zones.

## Figures and Tables

**Table 1:** Generalized linear model (GLM) results showing how independent variables influence the number of collected mosquitoes.

Explanatory Variables	β	±SE	Cl 2.5%	Cl 97.5%	z Value	*p* Value
(Intercept)	3.526	0.561	2.40	4.63	6.290	<0.001 ***
Temperature	−0.099	0.019	−0.138	−0.060	−5.210	<0.001 ***
Wind conditions	−0.129	0.065	−0.262	0.005	−1.991	0.047 *
Study Site (Ref: Site A)						
Site B	0.852	0.481	−0.077	1.817	1.771	0.077
Site C	1.080	0.478	0.168	2.032	2.261	0.024 *
Site D	1.465	0.460	0.601	2.380	3.185	0.001 **
Site E	2.092	0.447	1.244	2.996	4.678	<0.001 ***
Site F	2.789	0.439	1.957	3.680	6.361	<0.001 ***
Site G	1.637	0.458	0.773	2.553	3.573	<0.001 ***
Site H	2.762	0.442	1.928	3.654	6.252	<0.001 ***
Site 1	1.440	0.415	0.663	2.272	3.465	0.001 ***
Site 2	0.070	0.429	−0.739	0.928	0.163	0.871
Site 3	0.197	0.428	−0.609	1.053	0.460	0.645
Site 4	0.731	0.421	−0.064	1.578	1.737	0.082
Site 5	0.534	0.423	−0.267	1.386	1.262	0.207
Site 6	1.231	0.416	0.446	2.070	2.956	0.003 **
Site 7	0.806	0.431	−0.003	1.666	1.871	0.061
Collection Year (Ref: 2013)						
2016	−1.948	0.317	−2.617	−1.287	−6.139	<0.001 ***
2017	−3.627	0.331	−4.315	−2.962	−10.965	<0.001 ***
Collection Month (Ref: May)						
June	−0.479	0.353	−1.117	0.159	−1.357	0.175
July	−1.844	0.339	−2.492	−1.206	−5.444	<0.001 ***
August	−1.584	0.334	−2.234	−0.943	−4.747	<0.001 ***
September	−3.558	0.409	−4.366	−2.764	−8.710	<0.001 ***
Gender (Ref: Female)						
Male	−0.887	0.264	−1.435	−0.335	−3.364	<0.001 ***
Interactions between Year (Ref: 2013) and Month (Ref: May)						
2016: June	0.675	0.440	−0.217	1.564	1.533	0.125
2017: June	1.639	0.415	0.823	2.458	3.946	<0.001 ***
2016: July	2.167	0.427	1.285	3.052	5.069	<0.001 ***
2017: July	2.743	0.413	1.909	3.586	6.642	<0.001 ***
2016: August	1.601	0.414	0.750	2.453	3.864	<0.001 ***
2017: August	1.673	0.408	0.861	2.491	4.100	<0.001 ***
2016: September	3.390	0.457	2.457	4.329	7.420	<0.001 ***
2017: September	4.026	0.466	3.104	4.959	8.634	<0.001 ***
Interactions between Month (Ref: May) and Insect Gender (Ref: Female)						
June: Male gender	−0.175	0.341	−0.872	0.518	−0.515	0.607
July: Male gender	0.229	0.333	−0.452	0.906	0.686	0.493
August: Male gender	0.817	0.333	0.135	1.496	2.452	0.014 *
September: Male gender	1.197	0.333	0.507	1.884	3.594	<0.001 ***

Deviance residuals: min = −2.1444; 1Q = −1.0263; median = −0.6284; 3Q = 0.2327; max = 3.2837. Theta: 0.7169, standard error (SD): 0.0575. Null deviance 1410.84 on 1035 degrees of freedom (df), residual deviance 945.81 on 1000 df. Significance symbols: 0.05 to 0.01 = “*”, 0.01 to 0.001 = “**”, <0.001 = “***”. Abbreviation as follows: Estimates (β), standard error (±SE) and confidence limit (Cl).

**Table 2:** Generalized linear model (GLM) results showing how collection site and year influenced the effective number of species (ENS).

Explanatory Variables	β	±SE	CI 2.5%	CI 97.5%	t Value	*p* Value
(Intercept)	−0.065	0.357	−0.835	0.58	−0.182	0.856
Collection Sites (Ref: Site A)						
Site B	0.442	0.427	−0.382	1.318	1.034	0.301
Site C	0.747	0.405	−0.019	1.589	1.847	0.065
Site D	0.747	0.405	−0.019	1.589	1.847	0.065
Site E	1.269	0.377	0.571	2.069	3.362	<0.001 ***
Site F	1.541	0.367	0.868	2.326	4.194	<0.001 ***
Site G	0.636	0.412	−0.149	1.489	1.543	0.123
Site H	1.598	0.367	0.925	2.383	4.350	<0.001 ***
Site 1	0.893	0.358	0.243	1.665	2.491	0.013 *
Site 2	0.343	0.368	−0.330	1.130	0.931	0.352
Site 3	0.180	0.372	−0.502	0.974	0.484	0.628
Site 4	0.553	0.364	−0.110	1.333	1.519	0.129
Site 5	0.256	0.370	−0.422	1.046	0.691	0.490
Site 6	0.737	0.361	0.082	1.513	2.044	0.041 *
Site 7	0.328	0.380	−0.374	1.134	0.863	0.388
Study Years (Ref: 2013)						
2016	−0.109	0.107	−0.319	0.102	−1.013	0.311
2017	−0.628	0.127	−0.881	−0.381	−4.932	<0.001 ***

Deviance residuals: min = −2.2229; 1Q = −1.1782; median = −0.1585; 3Q = 0.6215; max = 2.9675. Null deviance 706.51 on 517 degrees of freedom (df), residual deviance 595.01 on 501 df. Significance symbols: 0.05 to 0.01 = “*”, <0.001 = “***”. Abbreviation as follows: Estimates (β), standard error (±SE) and confidence limit (Cl).

**Table 3:** List of mosquito species and groups collected during the study in alphabetical order. The table contains the number of individuals from each identified taxon, followed by the percentage (%) of female mosquitoes. Six collection sites were sampled during 2013, seven sites in 2016 and 15 collection points in 2017. The mean number of mosquitoes caught during a collection event was 6.41 in 2013, 3.78 in 2016 and 2.53 in 2017.

Species	2013		2016		2017	
	Total	% Female	Total	% Female	Total	% Female
Unspecified	15	93.33	21	85.71	11	90.91
*Aedes (Aedes) cinereus geminus*	45	66.67	109	49.54	108	57.41
*Aedes (Aedimorphus) vexans* (Meigen, 1830)	4	75.00	40	67.50	31	64.52
*Aedes (Ochlerotatus) annulipes* group	106	94.34	74	48.65	133	75.19
*Aedes (Ochlerotatus) cataphylla* Dyar, 1916	0	NA	7	85.71	29	86.21
*Aedes (Ochlerotatus) communis* (de Geer, 1776)	138	95.65	19	42.11	23	73.91
*Aedes (Ochlerotatus) diantaeus* Howard, Dyar and Knab, 1913	1	0.00	0	NA	0	NA
*Aedes (Ochlerotatus) excrucians* (Walker, 1856)	16	81.25	6	66.67	8	75.00
*Aedes (Ochlerotatus) flavescens* (Müller, 1764)	1	100.00	2	50.00	0	NA
*Aedes (Ochlerotatus) intrudens* Dyar, 1919	106	95.28	3	100.00	2	100.00
*Aedes (Ochlerotatus) leucomelas* (Meigen, 1804)	0	NA	2	50.00	7	85.71
*Aedes (Ochlerotatus) pullatus* (Coquillett, 1904)	0	NA	1	100.00	0	NA
*Aedes (Ochlerotatus) punctor/ punctodes*	44	75.00	38	52.63	76	67.11
*Aedes (Ochlerotatus) sticticus* (Meigen, 1838)	0	NA	36	94.44	26	84.62
*Anopheles (Anopheles) claviger* (Meigen, 1804)	3	0.00	0	NA	1	100.00
*Anopheles (Anopheles) maculipennis* complex	4	25.00	3	33.33	2	100.00
*Coquillettidia (Coquillettidia) richiardii* (Ficalbi, 1889)	11	72.73	2	100.00	10	80.00
*Culex (Culex) pipiens/torrentium*	151	30.46	163	43.56	202	33.17
*Culex (Neoculex) territans* Walker, 1856	9	22.22	27	33.33	4	0.00
*Culiseta (Culicella) ochroptera* (Peus, 1935)	0	NA	3	66.67	1	100.00
*Culiseta (Culiseta) annulata* (Schrank, 1776)	0	NA	0	NA	6	16.67
Total	654		556		680	

## Data Availability

The data presented in this study is openly available in FigShare at DOI: 10.6084/m9.figshare.14198951 (accessed on 12 March 2021). The dataset can also be obtained from the corresponding author.
